# Anxiety and Perceived Risk in Red Cross Volunteer Personnel Facing the Coronavirus Disease 2019 Pandemic

**DOI:** 10.3389/fpsyg.2021.720222

**Published:** 2021-10-25

**Authors:** José Antonio Ponce-Blandón, Victor Manuel Jiménez-García, Rocío Romero-Castillo, Manuel Pabón-Carrasco, Nerea Jiménez-Picón, Roger Calabuig-Hernández

**Affiliations:** ^1^Red Cross Nursing School, University of Seville, Seville, Spain; ^2^International Federation of Red Cross, Ecuador Country Office, Quito, Ecuador; ^3^Faculty of Nursing, Physiotherapy and Podiatry, University of Seville, Seville, Spain

**Keywords:** anxiety, perceived risk, COVID-19 pandemic, cooperation, humanitarian aid, volunteer staff

## Abstract

In the current situation of sanitary emergencies, humanitarian organizations and their volunteers are playing an important role in the coronavirus disease 2019 (COVID-19) pandemic. A study is proposed that includes a network of volunteers who perform humanitarian activities during the COVID-19 pandemic to assess anxiety, perceived risk, and response behaviors and to explore their relationship with sociodemographic variables. For data collection, an online questionnaire was developed through the Google Forms® platform, where the perceived risk, anxiety, and behavioral responses of the general population to the Influenza A (H1N1) pandemic were assessed. The survey presented is a modified version of that survey adapted for COVID-19. This adaptation was endorsed by an experts committee made up of the health chief of the Ecuadorian Red Cross, the focus point of operations from the International Federation of the Red Cross in Ecuador, and a member from the Health Unit of the Americas Regional Office of the International Federation of the Red Cross. A significant relationship has been shown between the job situation and perceived risk and anxiety, being the staff who worked full time away from home, which was exposed to greater risk and anxiety. Both perceived risk and perceived anxiety are very high (according to a 5-point Likert scale). Knowing these data from this first-line personnel will allow adopting measures that could be beneficial for stress management and, therefore, contribute to the well-being and support of these humanitarian and volunteer organizations in the worldwide response to COVID-1 9.

## Introduction

The epidemic caused by the coronavirus disease 2019 (COVID-19) in China was a threat to global health and represents the largest outbreak of atypical pneumonia since that of the Severe Acute Respiratory Syndrome (SARS) in 2003 (Wang et al., 2020a; WHO, 2020). Few weeks after the initial outbreak, the total number of cases and deaths surpassed those of SARS (Hawryluck et al., 2004). The outbreak manifested itself for the first time, in December 2019, when it was discovered that some groups of pneumonia cases of unknown etiology were associated with the exposure epidemiologically linked to a seafood market and untracked exposures in the city of Wuhan, province of Hubei (Nishiura et al., 2020). Since then, the number of cases has been increasing exponentially both in and outside Wuhan, extending to the 34 Chinese regions by January 30, 2020 (Wang et al., 2020b). That same day, the WHO declared that the COVID-19 outbreak was a global health problem classified as an international emergency (Mahase, 2020).

In addition to physical harm, COVID-19 also has a severe impact on mental health. This impact is seen on the general population, which shows behaviors related to the anxiety caused by the significant shortage of medical masks and hydroalcoholic gel in China. An important mental health burden is identified in the Chinese population during the COVID-19 outbreak, people who spent too much time thinking about the outbreak and health workers with a high risk of presenting psychological problems (Huang and Zhao, 2020).

There are several studies assessing perceived anxiety in the health personnel who performed tasks in pandemics (Wu et al., 2009). Consequently, the health personnel who performed tasks related to the Middle-East Respiratory Syndrome (MERS) had the highest risk of presenting symptoms of traumatic stress disorder, even after 2 months (Lee et al., 2018).

The health workers who responded to the spread of COVID-19 reported high rates of depression and anxiety symptoms, and insomnia, and anguish (Lai et al., 2020). It was discovered that most of these workers feel that they worked undertaking a significant personal risk, in a setting about which they are not properly informed, playing a role for which they are not sufficiently trained. Each worker must better understand the setting and the importance of their personal role in these environments (Balicer et al., 2006a). Despite the common mental health disorders among patients and health workers, most of these professionals working in isolation units and hospitals do not receive any training to provide mental healthcare (IFRC, 2020a).

In the current situation of sanitary emergency, humanitarian organizations and their volunteers play an important role in the COVID-19 pandemic, providing services to those affected (IFRC, 2020a). Armed conflicts, natural disasters, and other emergencies have an immense impact on long-term mental health and psychological well-being, including the volunteers who work in the entire context (von Keudell et al., 2016). Hence, the importance of preserving the well-being of these volunteers, taking their mental health into consideration (IFRC, 2020b). There are studies assessing perceived anxiety in the health personnel who performed tasks in pandemics (Lee et al., 2018), but not in volunteer personnel from humanitarian organizations who perform tasks in pandemics. It becomes necessary to study the psychological impact on the mental health of the medical workers and the communities to prepare for the response of a population to a disaster (von Keudell et al., 2016).

The perceived risk among the public health workers and the humanitarian-aid volunteers is associated with several factors, which are peripheral to the real peril of this event (IFRC, 2020b). These modifiers of the risk perception and the knowledge gaps identified to act as barriers to responding to the pandemic and must be specifically addressed to allow for an effective public health response (Balicer et al., 2006b).

In the general population, the uncertainty with which an outbreak of this magnitude is confronted becomes especially pertinent. Most of the population classifies the psychological impact as moderate or severe, with depression, anxiety, or stress being more prevalent (Wang et al., 2020b). There are tools to assess and predict health behaviors (such as depression, anxiety, and perceived risks) based on the Protection Motivation Theory (Conner and Norman, 2005) and on the Model of Health Beliefs (Champion and Skinner, 2008), which have been used in different studies (Brug et al., 2004; Bults et al., 2011).

The level of perceived risk related to the disaster will be influenced by the level of awareness and knowledge of a person (Commodari, 2017). The governmental programs aimed at enhancing such knowledge and awareness exert an influence on the perceptions of people and can help a society to be better prepared and to have greater control of a disaster situation. However, such programs can also have detrimental effects, as a result of the increase in the anxiety levels of individuals (Wu et al., 2009).

Given the above results, a study is proposed that includes a network of volunteers who perform humanitarian activities during the COVID-19 pandemic to assess anxiety, perceived risk, and response behaviors and to explore their relationship with sociodemographic variables. The data obtained gave us information on the psychological well-being of its volunteers, contributing to maintaining these personnel and recruiting new volunteers, thus, ensuring the quality of the service provided (Council of the Delegates of the International Red Cross Red Crescent Movement, 2019; IFRC, 2020b).

## Materials and Methods

### Study Design and Participants

A cross-sectional, observational, and descriptive study is conducted to assess the level of anxiety, perceived risk, and behavioral responses in the face of the COVID-19 pandemic with a group of intervening volunteers from the Ecuadorian Red Cross who are to perform humanitarian tasks.

The study population consisted of volunteers and hired intervening personnel from the Ecuadorian Red Cross that was imminently going to execute operational activities related to the pandemic in its entire Territorial Network. The population was accessed through the participants of the “Induction plan: Handling of Personal Protective Equipment (PPE) and application of protocols to the activities of operatives in the territorial network” that was developed by the Ecuadorian Red Cross, where it was foreseen that the institutional humanitarian personnel would receive information by means of a virtual platform to theoretically level up knowledge on the adequate use and handling of PPE in the response to the pandemic.

The following inclusion criteria were used to participate in the sample:

- Being intervening personnel belonging to cities defined as of immediate intervention, where five branches were located: Guayaquil, Quito, Babahoyo, Portoviejo, and Cuenca.
- Not belonging to groups vulnerable to COVID-19 or living in the same household with people from the vulnerable group, which, according to the WHO criteria, are as follows: individuals over 65 years old, immuno-depressed patients, and people with concomitant diseases, such as cardiovascular or respiratory conditions, cancer, and cerebrovascular diseases.

In these five aforementioned branches, there are 312 intervening volunteers available, selecting those who participated in the first phase of the plan, where all were screened by the Ecuadorian Red Cross through an affiliation interview to verify and responsibly declare, among other issues, that no intervening volunteer belonged to any risk group vulnerable to COVID-19 or lived in the same household with people belonging to these groups. In the first phase, the participants were 115 volunteers. A sample size calculation was performed with a 95% CI and an expected frequency of 50%, the minimum sample size being 89 subjects. Finally, 90 subjects were recruited in this study.

### Data Collection

For data collection, an online questionnaire was developed through the Google Forms® platform, where the perceived risk, anxiety, and behavioral responses of the general population to the Influenza A (H1N1) pandemic were assessed (Bults et al., 2011). This scale has a good reliability value (KMO 0.94) with a Cronbach's alpha coefficient of 0.85. The survey presented is a modified version of that survey adapted for COVID-19. This adaptation was endorsed by an experts committee made up of the health chief of the Ecuadorian Red Cross, the focus point of operations from the International Federation of the Red Cross in Ecuador, and a member from the Health Unit of the Americas Regional Office of the International Federation of the Red Cross.

The sociodemographic variables used in the descriptive study were as follows: gender, age, type of housing, marital status, schooling level, having pets, and work situation. The questionnaire also assessed variables referring to the evaluation of the information sources, and quantity and quality of the information received about COVID-19, and also an assessment of knowledge on COVID-19. Of all these, the following are considered as independent variables for the exploratory hypotheses: gender, type of housing, marital status, and information sources.

The data corresponding to the assessment of anxiety, perceived risk, physical symptoms, and behavioral responses were collected through the 26-item questionnaire on the severity level perceived, concern, thoughts, fear, psychosomatization, and habitual practices by using a 5-point Likert-type scale. This scale structures these 26 items in four categories: anxiety, perceived risk, physical symptoms, and behavioral responses. The values of these categories were considered as dependent variables in the exploratory hypotheses.

### Statistical Analysis

Data analysis was performed with the Epi Info version 7: Centers for Disease Control and the World Health Organization and IBM SPSS version 24: International Business Machines Corporation tools. For the descriptive analysis of the qualitative variables, the relative and absolute frequencies were calculated with 95% CI, whereas for the quantitative variables a numerical summary was conducted by calculating the centralization and dispersion measures.

To contrast the exploratory hypothesis, a bivariate analysis was performed between the set of sociodemographic variables/information sources and the variables of anxiety/perceived risk/physical symptoms/behavioral responses; all the dependent variables were recorded. The answers given by all the individuals to the items corresponding to each dependent variable were added up, calculating the answer total mean value of each. The individuals who obtained an average below this total mean value in the items of this variable were considered as a “low or very low” value, and those who obtained an average above the total mean value of the dependent variable were considered as a “high or very high” value, by using a methodology for recoding and for establishing cut-off points, very similar to that of other studies (Ragland, 1992; Maxwell and Delaney, 1993; Cumsille and Bangdiwala, 2000). The independent variable related to the information media was dichotomized, grouping official, information sources in one group and non-official information sources in another.

### Ethical Considerations

The study was reviewed and approved by the Ethics Committee for the Red Cross Nursing School of the University of Seville. The privacy of participants was preserved so that the questionnaire was completely anonymous, including informed consent for their participation. Throughout the data collection process, the ethical principles for medical research in human beings described in the latest review of the Declaration of Helsinki conducted in Brazil were applied (Asociación Médica Mundial, 2013). Authorization was obtained from the International Federation of the Red Cross and the Ecuadorian Red Cross.

## Results

Of the 312 volunteers from the five branches of the Ecuadorian Red Cross intended to receive the initial training for the response to COVID-19, the final convenience sample was made up of 90 participants, which corresponded to the definite number of volunteers who voluntarily answered the online questionnaire before the first session of the training program on the virtual platform devised for such purpose. Regarding the sociodemographic description of the sample, 55.5% were women ([95% CI: 44.7–66] *n* = 50), and the mean age of the participants in the sample was 29.5 years old (SD: 9.2). In relation to the type of housing, 56.7% of the participants ([95% CI: 45.8–67.1] *n* = 51) live in a house with a garden or a yard. About 24.4% of the participants were married or lived with a partner ([95% CI: 16–34.6] *n* = 22) and 46.7% of them had completed high school ([95% CI: 36.1–57.5] *n* = 42). Regarding the work situation, 20% of the participants were working full time outside their homes ([95% CI: 12.3–29.7] *n* = 18). The detail of all the results from the sociodemographic descriptive analysis of the study participants is given in Table 1.

**Table 1:** Sociodemographic results of the sample.

**Variables**	**Items**	**Absolute frequency (*n*)**	**Percentage (%) CI 95%**
Sex	Men	40	44.4% (33.9–55.3)
	Women	50	55.5% (44.7–66.0)
Type of living place	Apartment with terrace or patio	5	5.6% (1.8–12.5)
	Apartment without terrace or patio	11	12.2% (6.3–20.8)
	House with garden	51	56.7% (45.8–67.1)
	House without garden	23	25.6% (16.9–35.9)
Marital status	Single	60	66.6% (55.9–36.3)
	Married or living together	22	24.4% (16–34.6)
	Separated, divorced, or widowed	8	8.9% (3.9–16.8)
Educational level	Basic education	2	2.2% (0.3–7.8)
	Secondary education	42	46.7% (36.1–57.5)
	Training cycle education	32	35.6% (25.7–46.4)
	University education	11	12.2% (6.3–20.8)
	Postgraduate: master or doctorate	3	3.3% (0.7–9.4)
Job situation	Full time from home	4	4.4% (1.2–10.9)
	Full time away from home	18	20% (12.3–29.7)
	Part time from home	14	15.6% (8.8–24.7)
	Part time away from home	5	5.5% (2.5–8.5)
	Unemployed	17	18.9% (11.44–28.5)
	Retired	1	1.1% (0.0–6.0)
	Student	31	34.4% (30.4–38.4)
Employment contract	Autonomous	24	26.7% (17.9–37.0)
	Public employee	7	7.8% (3.9–15.4)
	Employment in private company	27	30% (20.8–40.6)
	Other situations	32	35.6% (25.7–46.3)

Regarding the perceived severity level imposed by COVID-19, 50% of the sample perceives it as “very high” ([95% CI: 39.3–60.7] *n* = 45). Concern for COVID-19 is “very high” in 44.4% ([95% CI: 33.9–55.3] *n* = 40) of the sample. In relation to the physical symptoms, 21.1% ([95% CI: 13.2–30.9] *n* = 19) of the sample refers to moderate stomach discomfort. Regarding the behavioral responses, frequent hand hygiene is always performed by 80% ([95% CI: 70.2–87.7] *n* = 72) of the sample; and, in relation to the use of masks, 82.2% of the sample ([95% CI: 72.7–89.4] *n* = 74) states using them at all times.

Regarding the efficacy level attributed to the preventive measures, that given to the use of masks before the state of alert is “high” in 55.6% ([95% CI: 44.7–66.0] *n* = 50) of the sample. The efficacy level attributed to the use of masks in the current time is “high” in 81.1% ([95% CI: 71.5–88.6] *n* = 73) of the sample. Regarding the level of confronting, 53.3% of the sample ([95% CI: 42.5–63.9] *n* = 48) states that the situation is worse than what was predicted (Table 2).

**Table 2:** Descriptive data on risk and perceived anxiety, physical symptoms, and behavioral responses to coronavirus disease 2019 (COVID-19).

	**Frequency (*****n)*** **Percentage % (95% CI)**				
**Perceived risk**	Very low	Low	Moderate	High	Very high
Degree of perceived severity	2 2.2 (0.3–7.8)	4 4.4 (1.2–10.9)	10 11.1 (5.5–19.5)	29 32.2 (22.7–42.9)	45 50 (39.3–60.7)
Risk of contracting the disease due to age previous pathologies	3 3.3 (0.7–9.4)	8 8.9 (3.9–16.7)	24 26.7 (17.9–37.0)	37 41.1 (30.8–51.9)	18 20 (12.3–29.7)
This disease is very harmful to me	0	7 7.8 (3.18–15.4)	17 18.9 (11.4–28.5)	29 30 (20.8–40.6)	39 43.3 (32.9–54.2)
Perceived susceptibility to getting sick	2 2.2 (0.3–7.8)	8 8.9 (3.9–16.8)	32 35.6 (25.7–46.3)	27 30 (20.8–40.6)	21 23.3 (15.1–36.4)
Possibility of getting infected	1 1.1 (0.0–6.0)	10 11.1 (5.5–19.5)	28 31.1 (21.8–41.7)	28 31.1 (21.8–41.7)	23 25.6 (16.9–35.8)
Possibility of infecting others	2 2.2 (0.3–7.8)	15 16.7 (9.6–26.0)	26 28.9 (19.8–39.4)	30 33.3 (23.7–44.0)	17 18.9 (11.4–28.5)
**Perceived anxiety**	Very low	Low	Moderate	High	Very high
Concern about COVID- 19	2 2.2 (0.3–7.8)	6 6.7 (2.5–13.9)	16 17.8 (10.5–27.3)	26 28.9 (19.8–39.4)	40 44.4 (33.9–55.3)
Fear of COVID-19	3 3.3 (0.7–9.4)	11 12.2 (6.3–20.8)	15 27.8 (18.8–38.2)	22 24.4 (16.0–34.6)	29 32.2 (22.7–42.9)
Frequency of thinking about COVID-19	5 5.6 (1.8–12.5)	25 27.8 (18.8–38.2)	21 23.3 (15.1–33.4)	26 28.9 (19.8–39.4)	13 14.4 (7.9–23.4)
**Physical symptoms**	Very low	Low	Moderate	High	Very high
Stomach discomfort	30 33.3 (23.7–44.0)	15 16.7 (9.6–26)	19 21.1 (13.2–30.9)	14 15.6 (8.8–24.7)	12 13.3 (7.1–22.1)
Sweat	29 32.2 (22.7–42.9)	16 17.8 (10.5–27.3)	22 24.4 (16.0–34.6)	12 13.3 (7.1–22.1)	11 12.2 (6.3–20.8)
Tremors	52 57.8 (46.9–68.1)	12 13.3 (7.1–22.1)	16 17.8 (10.5–27.3)	8 8.9 (3.9–16.8)	2 2.2 (0.3–7.8)
Tension	31 34.4 (24.7–45.2)	17 18.9 (11.4–28.5)	22 24.4 (16.0–34.6)	11 12.22 (6.3–20.8)	9 10 (4.7–18.1)
**Behavioral responses**	Never	Almost never	Sometimes	Almost always	Always
I practice frequent hand washing	1 1.1 (0.0–6.0)	0	2 2.2 (0.2–7.8)	15 16.6 (9.6–26)	72 80 (70.2–87.7)
I stay home	3 3.3 (0.7–9.4)	8 8.8 (3.9–16.7)	3 3.3 (0.7–9.4)	17 18.8 (11.4–28.5)	59 65.5 (54.8–75.2)
I always use a mask	0	1 1.1 (0.0–6.0)	2 2.2 (0.2–7.8)	13 14.4 (7.9–23.4)	74 82.2 (72.7–89.4)
I avoid crowded places	1 1.1 (0.0–6.0)	3 3.3 (0.7–9.4)	7 7.7 (3.1–15.3)	16 17.7 (10.5–27.2)	63 70 (59.4–79.2)

**Figure 1:**
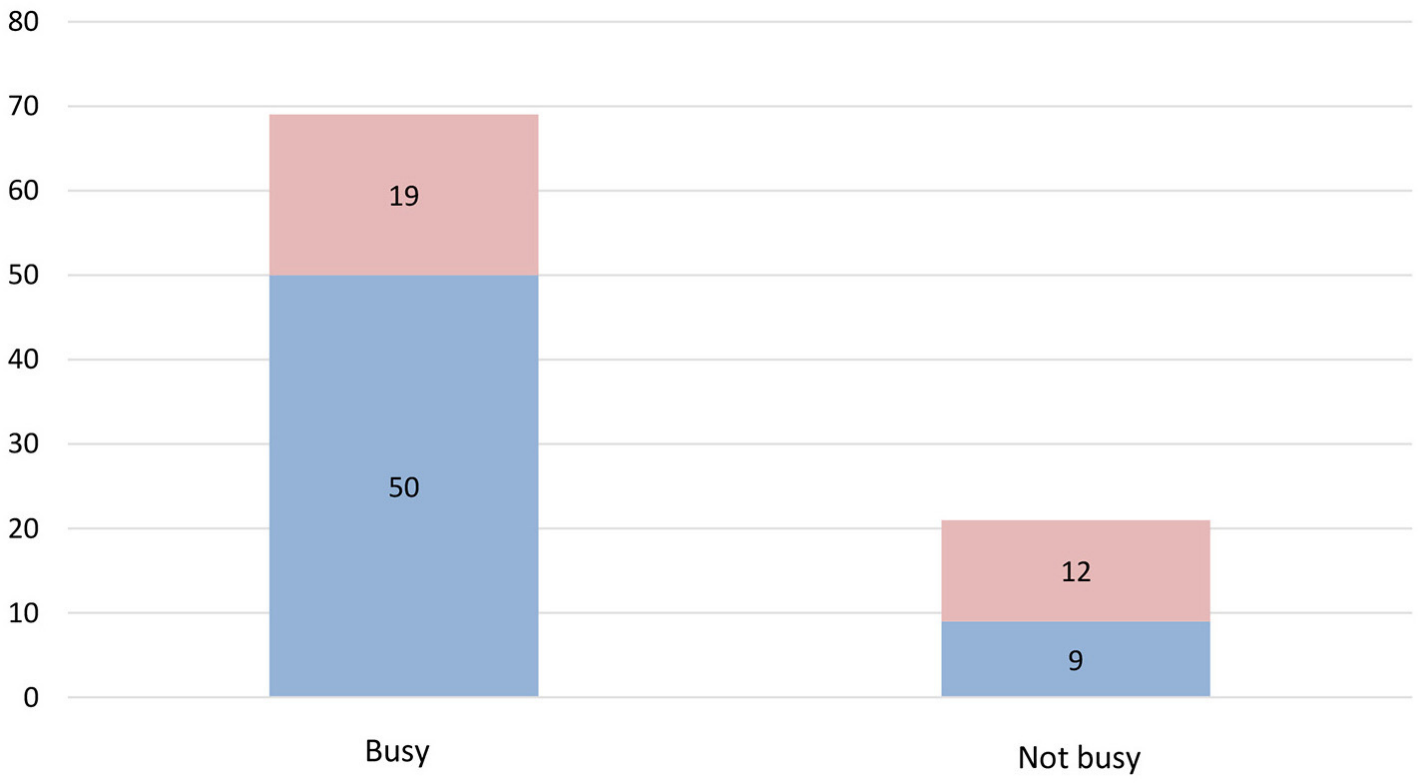
Job situation-perceived anxiety.

**Figure 2:**
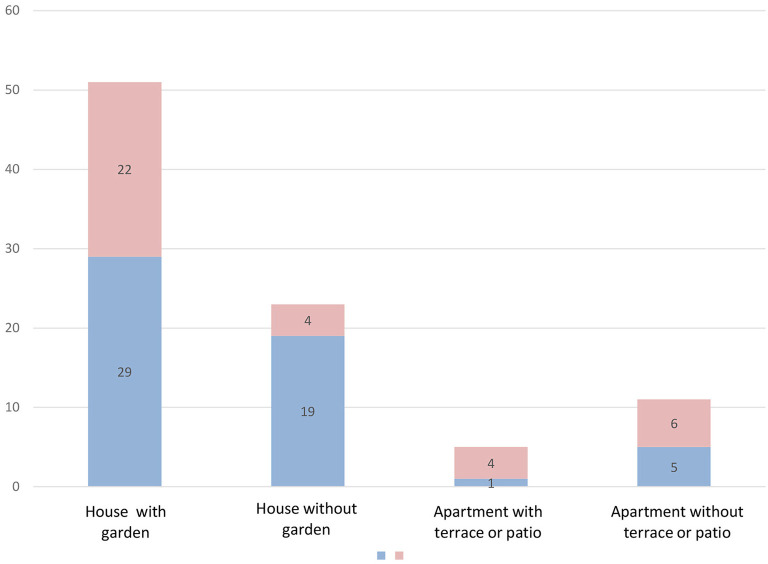
Type of living place-behavioral responses.

A statistically significant difference is observed between perceived anxiety and type of living place and job situation (*p* = 0.029 and *p* = 0.0124, respectively) (Figure 1). No significant results were found in the high values for perceived risk when contrasting them with gender, marital status, job situation, and type of housing (*p* = 0.924, *p* = 0.508, *p* = 0.348, and *p* = 0.211, respectively). No significant results were found in the high values for behavioral responses when contrasting them with gender, marital status, and job situation (*p* = 0.194, *p* = 0.106, and *p* = 0.677, respectively). However, when comparing the values of the type of housing with the behavioral responses, statistically significant differences were found (*p* = 0.024) (Figure 2), since levels of adequate behavioral responses to the COVID-19 were more frequently found in participants who lived in houses (with a garden or a yard, 56.8%; and without them, 82.6%) against those who lived in apartments (with a balcony, a terrace or a yard, 20%; and without them, 45.4%). No significant results were found in the high values for physical symptoms when contrasting them with gender, marital status, job situation, and type of housing (*p* = 0.386, *p* = 0.316, *p* = 0.854, and *p* = 0.811, respectively) (Table 3). The variables perceived anxiety, perceived risk, behavioral responses, and physical symptoms did not have a significant relationship with the sources of information (official and unofficial) (Table 4).

**Table 3:** Bivariate analysis between sociodemographic variables and anxiety, perceived risk, physical symptoms, and behavioral responses.

**Bivariate analysis**	**Relative risk**	**Statistical value, degrees of freedom, statistical significance**
Sex-perceived anxiety	0.8 (0.6–1.3)	*x*^2^ = 0.4; df = 1; *p* = 0.502
Sex-perceived risk	0.9 (0.7–1.4)	*x*^2^ = 0.09; df = 1; *p* = 0.924
Sex-physical symptoms	1.2 (0.8–1.6)	*x*^2^ = 0.750; df = 1; *p* = 0.386
Sex-behavioral responses	0.7 (0.5–1.1)	*x*^2^ = 1.668; df = 1; *p* = 0.194
Type of living place-perceived anxiety	N/A	*x*^2^ = 4.54; df = 3; *p* = 0.029^*^
Type of living place-perceived risk	N/A	*x*^2^ = 4.51; df = 3; *p* = 0.211
Type of living place-physical symptoms	N/A	*x*^2^ = 0.959; df = 3; *p* = 0.811
Type of living place-behavioral responses	N/A	*x*^2^ = 9.41; df = 3; *p* = 0.024^*^
Marital status-perceived anxiety	N/A	*x*^2^ = 2.548; df = 2; *p* = 0.280
Marital status-perceived risk	N/A	*x*^2^ = 1.353; df = 2; *p* = 0.508
Marital status-physical symptoms	N/A	*x*^2^ = 2.301; df = 2; *p* = 0.316
Marital status-behavioral responses	N/A	*x*^2^ = 4.48; df = 2; *p* = 0.106
Job situation-perceived anxiety	1.7 (1.0–2.8)	*x*^2^ = 6.25; df = 1; *p* = 0.0124^*^
Job situation-perceived risk	1.2 (0.8–1.9)	*x*^2^ = 0.878; df = 1; *p* = 0.348
Job situation-physical symptoms	1.1 (0.3–3.6)	*x*^2^ = 0.033; df = 1; *p* = 0.854
Job situation-behavioral responses	1.02 (0.9–1.1)	*x*^2^ = 0.173; df = 1; *p* = 0.677
Educational level-perceived anxiety	0.7 (0.5–1.04)	*x*^2^ = 2.91; df = 1; *p* = 0.088
Educational level-perceived risk	1.08 (0.7–1.5)	*x*^2^ = 0.231; df = 1; *p* = 0.630
Educational level-physical symptoms	0.41 (0.1–1.2)	*x*^2^ = 2.73; df = 1; *p* = 0.097
Educational level-behavioral responses	0.97 (0.9–1.05)	*x*^2^ = 0.392; df = 1; *p* = 0.530

* *Statistically significant p < 0.05*.

**Table 4:** Bivariate analysis between information sources and anxiety, perceived risk, physical symptoms, and behavioral responses.

	**Frequencies (*n*)**	**Relative risk (95% CI)**	**Statistical value, degrees of freedom, statistical significance**
Source of information-perceived anxiety	Official sources 60% (29)	1.0 (0.7–1.5)	*x*^2^ = 0.099; df = 1; *p* = 0.753
	Unofficial sources 57% (24)		
Source of information-perceived risk	Official sources 60.4% (29)	1.2 (0.8–1.8)	*x*^2^ = 0.984; df = 1; *p* = 0.321
	Unofficial sources 50% (21)		
Source of information-behavioral responses	Official sources 66.6% (32)	1.2 (0.8–1.8)	*x*^2^ = 1.905; df = 1; *p* = 0.168
	Unofficial sources 52.4% (22)		
Source of information-physical symptoms	Official sources 54.1% (26)	0.8 (0.6–1.1)	*x*^2^ = 1.458; df = 1; *p* = 0.227

The level of perceived risk is explained by 29% by the information received, highlighting the most explanatory variables—the accessibility and quantity of information received by the media (*p* = 0.003 and *p* = 0.0008, respectively) and the accessibility, quality, quantity, and utility received from official sources (*p* = 0.034, *p* = 0.015, *p* = 0.031, and *p* = 0.018, respectively). The level of perceived anxiety is explained only in 18% by this set of variables, highlighting the explanatory variables—the quality of the information (*p* = 0.0008) and the usefulness of the information (*p* = 0.016) received from official sources. The level of behavioral responses is explained by 22%, highlighting the amount of information received from the media (*p* = 0.018) and the accessibility and quality of the information received from official sources (*p* = 0.002 and *p* = 0.004, respectively). Finally, the level of physical symptoms is explained by only 13%, highlighting the significant use of the information provided by official sources (*p* = 0.0128) (Table 5).

**Table 5:** Regression model.

	**Information supplied by the media Significance level and coefficient in the regression model (SD included)**				**Information supplied by official sources Significance level and coefficient in the regression model (SD included)**				**Correlation coefficient**	***F*-Statistic**
	**Accessibility**	**Quality**	**Quantity**	**Utility**	**Accessibility**	**Quality**	**Quantity**	**Utility**		
Perceived risk	*p* = 0.003^*^ 0.496 (0.164)	*p* = −0.226 0.207 (0.170)	*p* = 0.0008^*^ −0.629 (0.182)	*p* = 0.789 0.048 (0.179)	*p* = 0.034^*^ −0.457 (0.213)	*p* = 0.015^*^ 0.598 (0.243)	*p* = 0.031^*^ 0.460 (0.210)	*p* = 0.018^*^ −0.472 (0.196)	0.29	4.178
Perceived anxiety	*p* = 0.453 0.128 (0.170)	*p* = 0.308 −0.181 (0.176)	*p* = 0.672 −0.080 (0.189)	*p* = 0.221 0.229 (0.186)	*p* = 0.0738 −0.400 (0.221)	*p* = 0.0008^*^ 0.874 (0.252)	*p* = 0.525 0.139 (0.218)	*p* = 0.016^*^ −0.500 (0.204)	0.18	2.226
Behavioral responses	*p* = 0.210 0.225 (0.178)	*p* = 0.997 −0.001 (0.185)	*p* = 0.018^*^ −0.477 (0.198)	*p* = 0.162 0.275 (0.195)	*p* = 0.002^*^ −0.733 (0.232)	*p* = 0.004^*^ 0.766 (0.264)	*p* = 0.087 0.395 (0.229)	*p* = 0.088 −0.368 (0.214)	0.22	2.921
Physical symptoms	*p* = 0.966 0.010 (0.230)	*p* = 0.743 0.078 (0.238)	*p* = 0.794 0.066 (0.255)	*p* = 0.369 0.227 (0.251)	*p* = 0.844 −0.059 (0.298)	*p* = 0.084 0.595 (0.340)	*p* = 0.749 0.095 (0.295)	*p* = 0.0128^*^ −0.700 (0.275)	0.13	1.533

* *Statistically significant*.

## Discussion

The study intended to assess the level of anxiety, perceived risk, and behavioral responses to the COVID-19 pandemic in a group of intervening volunteers from the Ecuadorian Red Cross, who were initiating their preparation to participate in response activities against the COVID-19 pandemic. The data were collected in the first wave, from April to June of 2020.

Regarding the behavioral responses, a clear strength of this study is observed, since data collection took place during the pandemic, in opposition to other studies conducted at times when the pandemic was based on hypothetical situations (Hong and Collins, 2006; Taylor et al., 2009; Kok et al., 2010).

Regarding its limitations, this is a descriptive and cross-sectional study, with a convenience sample made up of volunteer personnel with a mean age of 29.5 years old, single, and with complete high school. Therefore, the results cannot be extrapolated to the general population, thus limiting external validity. It is a small sample that may be unrepresentative, but it met the minimum necessary sample size. Despite this, the sample turns out to be interesting, as there are few studies addressing the mental health and psychological aspects of intervening volunteers who are to perform humanitarian tasks in the face of a pandemic. The existing studies that address mental health and during COVID-19 pandemic psychological aspects during the COVID-19 pandemic focus on the general population (Galea et al., 2020; Liu et al., 2020), the patients (Lima et al., 2020), and the health personnel (Wu et al., 2009; Min et al., 2018; Lai et al., 2020; Pappa et al., 2020). Understanding the mental health response after a public health emergency might help the communities to prepare for the response of a population to a disaster (Das et al., 2020; Rajkumar, 2020).

Another limitation is the fact that the questionnaire used was adapted from a questionnaire specifically designed for the H1N1 pandemic (Bults et al., 2011). Nevertheless, an adaptation effort was made by a group of experts, who found many common elements between the H1N1 and the COVID-19 pandemics, which result in the non-previsibility of many biases caused by the validity of the instrument employed.

The exploratory analysis performed was bivariate; effectiveness might be increased by conducting a multivariate analysis. Nevertheless, the study object had an exploratory nature, allowing for the establishment of relationships between dependent and independent variables.

Regarding the dichotomization of continuous variables, it becomes necessary to discuss the possibility of substantially modifying the relationships between dependent and independent variables (Cumsille and Bangdiwala, 2000). Some authors suggest that there can be underestimation or underestimation biases about the association (Maxwell and Delaney, 1993). However, it seems that these biases are much more likely when the analyses are based on multiple linear regression models or when the logistic regression models are applicable (Cumsille and Bangdiwala, 1996), situations that do not apply to this study. Consequently, considering that the categorization of the continuous variables has allowed the researchers to avoid the strong assumptions required by these models about the relationship between the variables and the risk assessment, and the “Likert” answer scale for each item consisted of only five points, and it does not seem probable that too much information has been lost to bias the results (Altman et al., 1994).

Regarding perceived risk, there is a “very high” assessment of the perceived severity level of COVID-19. Similarly, regarding the perceived anxiety variable, there is a “very high” assessment in relation to concern about COVID-19. These are conclusions similar to that of another study in which the prevalence of depression in health personnel during the COVID-19 pandemic is analyzed (Pappa et al., 2020).

Most of the participants obtain information through social networks since the Internet facilitates access to information (Balicer et al., 2006b). Nevertheless, we have witnessed a massive infodemic with the audience being bombarded with a large amount of information, much of which is not scientifically correct (Naeem and Bhatti, 2020), and where the social networks play an important role in the dissemination of fake news (Ahmad and Murad, 2020; Al Jazeera, 2020), leading to confusion and exasperation in the population (Pew Research Center, 2020). Institutions, such as the International Federation of Library Associations (IFLA), have developed tools on how to detect fake news (IFLA, 2016). The websites of the official public health organizations considered as the best-quality online information source on COVID-19 (Conner and Norman, 2005) remain in this study as the third most used information source, which concedes major responsibility to the governments in relation to general interest sanitary and public health recommendations (Ministerio de Sanidad C y BS, 2020).

Despite the popularity and accessibility of the Internet, no significant association is found between using the Internet as an information source on COVID-19 and the behavioral responses, a result that coincides with a study-relating information source and self-confidence to face COVID-19 (Ajzen, 2002; Galea et al., 2020).

These measures coincide with those recommended by the WHO, where the importance of combining them to enhance their effectiveness is emphasized (bin-Reza et al., 2012). Other studies corroborate the importance of using masks (Cowling et al., 2010; MacIntyre and Chughtai, 2020).

No significant differences are appreciated regarding the perception of anxiety among individuals of different genders (78), which contrast other studies where a significant difference is indeed seen regarding gender during the COVID-19 pandemic or in the H1N1 pandemic, where the most concerned and anguished population segments due to the pandemic were women and aged individuals, more prone than others to adopt some avoidance conducts (Champion and Skinner, 2008; Lau et al., 2010; Taglioni et al., 2013).

Significant differences are appreciated between the type of housing and the behavioral responses adopted. The findings of this study represent an essential first step to understand if housing directly affects the adoption of adequate behavioral responses, since levels of adequate behavioral responses to the COVID-19 pandemic were more frequently found in participants who lived in houses, against those who lived in apartments.

No significant differences are established between marital status and anxiety or perceived risk. One of the reasons can be the reduced sample, though it might be expected that people who face the pandemic alone without a partner or with social distancing can present higher anxiety levels (Galea et al., 2020; Giallonardo et al., 2020). According to Elbay et al. (2020), the level of anxiety was mainly associated with the profile: young, single, with little work experience, and with work in the front line. The increase in weekly working hours, the greater number of patients diagnosed with COVID-19, a lower level of support from their reference people, less logistical support, and less feeling of competence during development were predictive factors of stress and anxiety development of tasks.

In conclusion, it was possible to assess anxiety, perceived risk, and response behaviors in the volunteer personnel during the COVID-19 pandemic. Both perceived risk and perceived anxiety are very high. However, the behavioral responses adopted are adequate.

A significant relationship has been shown between the behavioral responses and type of housing since levels of adequate behavioral responses were found in individuals who lived in houses against those who lived in apartments. Additionally, the relationship between the job situation and perceived risk and anxiety, being the staff who worked full time away from home, was exposed to greater risk and anxiety. Living in a house with open spaces, such as patios and terraces, was a protective factor for mental health during the months of home confinement. While the increase in the number of working hours and the full-time shift on the front line of the pandemic were factors that favored stress and perceived anxiety.

Knowing these data from this first-line personnel will allow adopting measures that could be beneficial for stress management and, therefore, contribute to the well-being and support of these humanitarian and volunteer organizations in the worldwide response to COVID-19, in order to help people and communities to prepare and respond to the global emergency. The most important measures would be focused on increasing knowledge and official information in this population since this increases their safety and reduces their stress level. It is also important to provide volunteers with material resources and clear recommendations. Unfortunately, this pandemic has been a new situation that has overtaken many of us and many recommendations have been changing. Among the measures that have been carried out in the Red Cross Organization itself, it is worth highlighting the courses on stress management aimed at intervening personnel. These courses have therapies and coping strategies for very stressful situations. There are exercises and drills of action in extreme situations. Although there is data of high satisfaction of the volunteers participating in these courses, we plan to obtain new learning results of the therapies to volunteers in our next study.

## Data Availability Statement

The original contributions presented in the study are included in the article/supplementary material, further inquiries can be directed to the corresponding author.

## Ethics Statement

The studies involving human participants were reviewed and approved by Ethic Committee of Red Cross Nursing School, University of Seville. The patients/participants provided their written informed consent to participate in this study.

## Author Contributions

JP-B and RC-H conceived the study. VJ-G, MP-C, and NJ-P collected the data and performed the analyses. RR-C wrote the manuscript. All the authors contributed to the article and approved the submitted version.

## Conflict of Interest

The authors declare that the research was conducted in the absence of any commercial or financial relationships that could be construed as a potential conflict of interest.

## Publisher's Note

All claims expressed in this article are solely those of the authors and do not necessarily represent those of their affiliated organizations, or those of the publisher, the editors and the reviewers. Any product that may be evaluated in this article, or claim that may be made by its manufacturer, is not guaranteed or endorsed by the publisher.
